# On the Increasing Frequency of Cancerous Diseases

**Published:** 1844-04

**Authors:** 


*On the Increasing Frequency of Cancerous Diseases.*—M. Tanchon remarks, "that there is reason to believe that the frequency of cancer is on the increase, more especially in large towns. Mr. Farr tells us that, in 1838, the number of deaths (in England, we believe) from this cause was 2,448, while in 1839 it rose to 2,691. My own inquiries lead to a similar conclusion; for, from a registry of cases of cancer of the uterus, I find that there were in—

| | Cases. | | Cases. |
|---|---|---|---|
| 1830 - - | 351 | 1833 - - | 498 |
| 1831 - - | 391 | 1834 - - | 436 |
| 1832 - - | 396 | 1835 - - | 508 |

"Cancer, like insanity, is of much more frequent occurrence in civilized than in barbarous countries. Long ago it was remarked in the East, that the disease was much oftener found in the Christian than in the Moslem population there. Fabricius Hildanus was of opinion that it was more frequent in temperate than very warm climates; and this opinion has certainly been confirmed by the experience of some recent observers. M. Hamon, an eminent veterinary surgeon, who has been attached to Mehemet Ali's service for 14 years, informs me that he has never seen a case of genuine cancer in the native or Fellah women, and not many even in the Turkish women of Egypt. M. Clotbey has made a similar remark; and M. Bac, surgeon-major of the second regiment of African *Chasseurs*, never met with a single case during the six years that he resided at Senegal. Again, M. Baudens, now the head surgeon at the Val de Grace, whose practice at Algiers was most extensive for a considerable number of years, did not meet with more than two or three instances altogether."

M. Tanchon closes his remarks with the following general conclusions:

"1. The frequency of cancerous diseases appears to be on the increase, and this frequency seems to be proportionate to the degree of civilization to which a people has attained.

"2. It is towards the decline of life, and more especially in the female sex, that the development of cancer is most to be dreaded; but early age is not entirely exempt from its attacks.

"3. Glandular organs, and those in particular which are the most susceptible of excitement, are most frequently the seat of the disease.

"4. The proximate cause of the disease appears to exist in every part of the system; but rather in the fluids than in the solids.

"5. When not induced by any external cause, it seems to result from a molecular organic modification or lesion of the part, occasioned by various causes, which as yet are but little known.

"6. In the actual state of our knowledge, the treatment of cancer can be little else but empirical, like that of syphilis, dartres, &c. Still there is reason to hope that we may yet learn how to arrest, if not actually to extirpate, the morbid process. In many cases, we well know that the disease is remarkably stationary for a great number of years, and indeed that its existence seems not much to interfere with the full prolongation of life. What are the circumstances favorable to inducing such an arrest, we indeed are almost wholly ignorant; but much may be expected from a careful watching of such cases by metropolitan surgeons, and others who have the opportunity of seeing many cases of cancerous maladies. The regulation of the diet is unquestionably of primary importance, and hence every means should be adopted to keep the digestive and assimilative organs in a sound healthy condition, in order that there may be a regular supply of wholesome chyle."—*Gazette des Hôpitaux.*—*Med. Chi. Rev.*

